# Spatial variations and determinants of bottle feeding among children aged 0–23 months in Ethiopia in 2019: A spatial and multi-level analysis

**DOI:** 10.1371/journal.pone.0311051

**Published:** 2024-09-26

**Authors:** Fantu Mamo Aragaw, Mehari Woldemariam Merid, Adugnaw Zeleke Alem, Dagmawi Chilot, Melaku Hunie Asratie, Anteneh Ayelign Kibret, Daniel Gashaneh Belay

**Affiliations:** 1 Department of Epidemiology and Biostatistics, Institute of Public Health, College of Medicine and Health Sciences, University of Gondar, Gondar, Ethiopia; 2 Department of Human Physiology, College of Medicine and Health Sciences, University of Gondar, Gondar, Ethiopia; 3 Department of Women’s and Family Health, School of Midwifery, College of Medicine and Health Sciences, University of Gondar, Gondar, Ethiopia; 4 Department of Human Anatomy, College of Medicine and Health Sciences, University of Gondar, Gondar, Ethiopia; University/College Library, ETHIOPIA

## Abstract

**Background:**

Bottle feeding should be avoided since it interferes with optimal breastfeeding and it causes diarrheal-related morbidity and mortality. Despite the WHO’s recommendation that children to avoid bottle feeding, it is still widely practiced in developing countries including our country, Ethiopia. Therefore, this study aimed to assess the spatial variations, and determinants of bottle feeding among children aged 0–23 months using the recent demographic and health survey data for Ethiopia.

**Methods:**

A secondary data analysis was conducted using the 2019 Ethiopian mini demographic and health survey data. The total weighted sample of 2067 children aged 0–23 months was included in this study. Spatial analysis was done to identify the hotspot areas of bottle feeding among children in Ethiopia. Multivariable multilevel logistic regression was used to identify predictors of bottle feeding. The spatial analysis was done using ArcGIS 10.7 and Sat Scan 9.6 software.

**Result:**

The prevalence of bottle feeding among children aged 0–23 months was 21.52% with 95% CI(19.80%, 23.34%). Age of the child from 6–11 months, and 12–23 months age, having secondary and above education [AOR = 2.09; 95%CI; 1.31, 3.32], being from middle and rich household [AOR = 2.14; 95%CI; 1.37, 3.34] and [AOR = 2.30; 95%CI; 1.46, 3.63], and twin birth [AOR = 8.06; 95%CI; 2.87, 22.58] were significant predictors of bottle feeding. Hotspot areas of bottle feeding were observed in Addis Ababa, Dire Dawa, Harari, and Afar regions of Ethiopia.

**Conclusion:**

Bottle feeding practice was found to be spatially clustered in Ethiopia. Education, wealth index, parity, and child’s age were significant predictors of bottle feeding. Hotspot areas of bottle feeding were observed in Addis Ababa, Dire Dawa, Harari, and Afar regions. Special attention should be directed towards mothers residing in hotspot areas, educated mothers, mothers of multiple births, and mothers from rich households through community education programs focused on child feeding practices to reduce the practice of bottle-feeding in Ethiopia.

## Background

Infant and young child feeding (IYCF) is a critical component of a child’s developmental care [1]. Appropriate infant and young child feeding including breastfeeding and complementary feeding are critical for the growth and development of children all over the world [2]. Bottle feeding should be avoided for infant and young child feeding because it interferes with optimal breastfeeding and appropriate complementary feeding [3].

Despite WHO recommendations that children avoid bottle feeding, it is still widely used in developing countries [4], which has been linked to diarrheal disease morbidity and mortality [4]. Bottle-feeding was practiced by 35.7% of children under the age of two in Namibia [5], and 37.9% in Indonesia [6]. Bottle feeding of infants has been strongly linked to poor breastfeeding conditions [7,8]. Bottles typically deliver milk in a manner that is thought to impede the transition to full breastfeeding [9].

Evidence have also indicated that bottle feeding causes a significant impact on both morbidity and mortality in children [10], particularly on diarrheal disease morbidity and mortality since bottles are prone to contamination and they are difficult to keep clean, particularly in developing countries with poor sanitation [3]. Bottle feeding increases the risk of infantile hypertrophic pyloric stenosis [11]. Evidence also indicated that bottle feeding has been linked to dental caries in children [12]. Bottle-fed infants are more likely to gain weight quickly than breast-fed infants, which can increase the risk of obesity [13–15].

According to several reports, bottle-feeding may cause the development of non-nutritive sucking habits, which may lead to malocclusion [16]. The sucking mechanism used in bottle-feeding differs significantly from that used in breastfeeding [17]. This distinction may predispose children who are bottle-fed for an extended period to malocclusion or other distinctive occlusion characteristics [18].

Evidence also suggests that mothers choose bottle feeding over breastfeeding due to a lack of sufficient breast milk, especially for those mothers having twin births [19,20]. Also, the age of mothers and children [4,21–24], educational status of women [4,22–25], occupational status of women [26], wealth index [4,23,25,26], parity [24], postnatal care [26], residence [26] were reported to have significant associations with bottle feeding practice.

According to the EDHS, the prevalence of bottle feeding in Ethiopia has increased from 13.5% in 2016 to 21.52% in 2019 [4]. Despite the increase in the magnitude of bottle-feeding practice, there is no updated evidence regarding the spatial distribution and determinants of bottle feeding in Ethiopia. As a result, the purpose of this study was to assess the spatial variation, and the individual and community level determinants of bottle feeding among children aged 0 to 23 months in Ethiopia, using recent nationally representative data. The findings of the study will help policymakers in Ethiopia implement measures to reduce bottle feeding and improve children’s optimal feeding practices.

## Methods

### Data sources and populations

This study was based on the 2019 Ethiopian Mini Demographic and Health Survey (EMDHS) dataset, which was the second EMDHS and the fifth DHS implemented in Ethiopia from March 21, 2019, to June 28, 2019. The 2019 EMDHS was conducted by the Central Statistical Agency in partnership with the Federal Ministry of Health and the Ethiopian Public Health Institute.

The sample used for the survey was stratified and selected using two stages. Firstly, a total of 305 enumeration areas (EAs) (93 in urban, 212 in rural) were chosen independently with a probability proportional to each EAs. Second, from the newly formed household listing, a fixed number of 30 households/clusters were selected with an equal probability of systematic selection. The detailed sampling procedures are available on the measure DHS website in the 2019 EMDHS report (https://www.dhsprogram.com). Data were obtained from the DHS website: www.dhsprogram.com by justifying the reason for requesting the data and after obtaining an approval letter from the DHS. The data is collected using standardized, structured, and pre-tested questionnaires that were developed in cooperation with the Demographic Health Survey (DHS) and customized to represent the Ethiopian population and health conditions [27]. The Kid’s record data set was used. We used the household record data set for this study. Finally, a total weighted sample of 2067 children were included in this study.

### Variables of the study

The dependent variable was bottle feeding described as drinking anything from a bottle with a nipple yesterday or the previous night among children aged 0–23 months, and it was categorized as "yes" if a child fed anything from a bottle with a nipple the night before, and "no" otherwise [28].

Socio-demographic and child-related characteristics were included as independent variables. Maternal age, marital status, maternal educational level, household head, household wealth, parity, sex of the child, child age, place of delivery, and multiple births were some of the individual-level factors included in this study. The wealth index was constructed using principal component analysis and then categorized as poorest (quintile 1), poorer (quintile 2), middle (quintile 3), richer (quintile 4), and richest (quintile 5) [29]. The wealth index scores were developed based on the number and kinds of consumer goods in a household, ranging from a television to a bicycle or car; housing characteristics such as the source of drinking water and toilet facilities; and flooring materials [30].

As community-level factors residence, community-level poverty, Community level of women’s education, and region were included. Regions were classified into three categories; Amhara, Oromia, Tigray, and SNNP regions were categorized as a large central region; Harari, Addis Ababa, and Dire Dawa were categorized as metropolitan regions, and the others (Somali, Gambelia, Afar, and Benishangul Gumuz region) were categorized as a small peripheral region [31,32].

After checking their distribution, the community poverty and literacy levels were divided as high or low, and the median value was used as the cut-off point for classification since the data was not normally distributed. The community poverty level was classified as high if the proportion of women from the two lowest wealth quintiles in a given community was greater than the median value and low if the proportion was below the median value [33]. The community level of women’s education was the proportion of women in the community with at least a primary level of education, classified as high (proportion of women greater than median national value) whereas low (proportion of women below-median national value) [34].

### Data management and analysis

After data was extracted editing, coding, and cleaning were performed. Data were weighted before statistical analysis using sampling weight (v005), primary sampling unit (v021), and strata (v022) to restore the survey’s representativeness and obtain valid statistical estimates. Both descriptive and analytic statistics were computed. We used a multilevel logistic regression analysis by assuming that each community has a different intercept and fixed coefficient, the clustered data nature as well as within and between community variations, with a random effect applied at the cluster level. Variables with p-values ≤0.2 in the bi-variable analysis were fitted in the multivariable model. Adjusted Odds Ratio (AOR) with a 95% Confidence Interval (CI) and p-value <0.05 in the multivariable model were used to declare a significant association with the outcome. The goodness of fit was checked using deviance. We calculated the variance inflation factor (VIF) for each predictor variable by conducting a pseudo-linear regression analysis.

### Spatial analysis

Spatial analysis was conducted in 2019 using the Geographic Information System (GIS) application to assess geographic variations of bottle-feeding cases among EDHS clusters. We received the GPS points in shape file format for the 2019 EDHS survey from the DHS office upon request. The location data (geographic coordinates) of each survey cluster were collected using Global Positioning System (GPS) receivers. To protect respondents’ privacy, GPS latitude/longitude positions for all survey groups were displaced by two kilometers in urban clusters and five kilometers in rural clusters [35].

We calculated the proportions of bottle-feeding cases for each cluster in the survey and then appended the latitude and longitude coordinates of the selected EAS in the 2019 EDHS survey. The spatial autocorrelation statistic (Global Moran’s I) was used to determine whether bottle feeding was dispersed, clustered, or randomly distributed in Ethiopia. The maximum peak distance where bottle feeding is more pronounced was determined using incremental spatial autocorrelation.

The Getis-Ord Gi* hot spot analysis is used to identify spatial clusters of high values (hot spot) and a spatial cluster of low value (cold spot) of bottle feeding among children in Ethiopia. To determine the statistical significance of clustering, the Z-score was computed, and the p-value was computed. A positive z-score with a P-value of 0.05 indicates clustering of statistically high hotspots; however, a negative z-score with a P-value of 0.05 indicates clustering of statistically low spots. A z-score close to zero indicates that there is no significant clustering.

Based on sampled clusters, the spatial interpolation technique is used to predict bottle feeding for unsampled areas. For unsampled cluster prediction, we employed a geostatistical ordinary Kriging spatial interpolation technique. Interpolation was done based on the assumption that spatially distributed objects are spatially correlated; in other words, things that are close together tend to have similar characteristics [36].

Bernoulli-based model spatial scan statistics were used to determine the geographical locations of statistically significant clusters for bottle feeding among children in Ethiopia. A likelihood ratio test statistic and the p-value were used for each potential cluster to determine whether the number of observed bottle feeding within the potential cluster was significantly higher than expected or not.

### Parameter estimation methods

Fixed effect estimates in the multilevel multivariable logistic regression model measure the association between the odds of bottle feeding of the individual- and community-level factors with a 95% confidence interval. The random effects were measured in terms of intra-class correlation (ICC), median odds ratio (MOR), and proportional change in variance (PCV) [37–39]. The median odd ratio(MOR) indicates the central value of the odd ratio between the highest and the lowest risk regions when two clusters are chosen at random. The ICC shows the differences between clusters in bottle feeding among children [40]. The PCV quantifies the proportion of total observed individual variation that can be attributed to between-cluster differences [41].

### Ethical consideration

All methods were carried out following the relevant guidelines of the DHS program. Informed consent was waived from the International Review Board of DHS program data archivists after the consent paper was submitted to the DHS Program, a letter of permission to download the dataset for this study. The dataset was not shared or passed on to other bodies and was anonymized to maintain its confidentiality.

## Results

### Socio-demographic characteristics of respondents

A total of 2067 children aged 0–23 months were included in the study. Nearly half of the mothers 957(46.31%) have not attended formal education. Around 890 (43.10%) of participants are from poor households. Half of the mothers belonged to the age group 25–34 years 1,035 (50.08%). Around three fourth 1,511(73.16) of the participants lived in rural areas (**Table 1**).

**Table 1:** Background characteristics of the study participants in a study of bottle feeding among children aged 0–23 months in Ethiopia, 2019 mini EDHS.

Variables	Categories	Weighted Frequency (n)	Weighted Percentage (%)
Age of women	15–24	649	31.40
	25–34	1,035	50.08
	35–49	382	18.52
Marital status	Married	1,948	94.28
	Not married	118	5.72
Women education status	No education	957	46.31
	Primary	823	39.85
	Secondary and higher	286	13.84
Parity	Primiparous	468	22.65
	Multiparous	965	46.74
	Grand multiparous	632	30.61
Place of delivery	home	922	44.65
	Institution	1,143	55.35
Household head	Male	1,795	86.89
	Female	270	13.11
Wealth index	Poor	890	43.10
	Middle	386	18.71
	Rich	789	38.19
Sex of child	Male	1,048	50.73
	Female	1,018	49.27
Age of child	0–5 months	553	26.79
	6–11 months	484	23.46
	12–23 months	1,028	49.75
Plurality of birth	Single	2,027	98.10
	Multiple	39	1.90
**Community level variables**			
Residence	Rural	1,511	73.16
	Urban	554	26.84
Community level poverty	Low	1,215	58.81
	High	851	41.19
Community level of women’s education	Low	975	47.20
	High	1,091	52.80
Regions	Tigray	152	7.37
	larger central	1,793	86.79
	small peripherals	192	9.30
	metropolis	80	3.91

### Prevalence of bottle feeding in Ethiopia

The prevalence of bottle feeding among children aged 0–23 months was 21.52% with 95% CI(19.80%, 23.34%) in Ethiopia.

### Random effect and model comparison

In the null model, the ICC indicated that 28% of the total variability for bottle feeding was due to differences between clusters while the remaining unexplained 72% of the total variability of bottle feeding was attributable to individual differences. The model with the lowest deviance (1760) was selected as the best-fitted model, which was model three (the model containing both individual and community-level variables) (**Table 2).**

**Table 2:** Model fitness and random effect measures.

Measure of variation	Null model	Model I	Model II	Model III
**VA**	1.33	1.31	0.92	1.23
**ICC**	0.28	0.28	0.22	0.27
**MOR**	2.97	2.95	2.47	2.86
**PCV (%)**	__	0.015	0.31	0.07
**Model fitness and comparison**				
**Deviance**	1958	1771	1910	1760
**Mean VIF**	__	1.56	1.50	1.76

ICC = Inter cluster corrolation cofficent, MOR = Median odds ratio, PCV = proportional change in variance, VIF = Variance inflation factor.

### Regional variation of bottle feeding in Ethiopia

**Fig 1:**
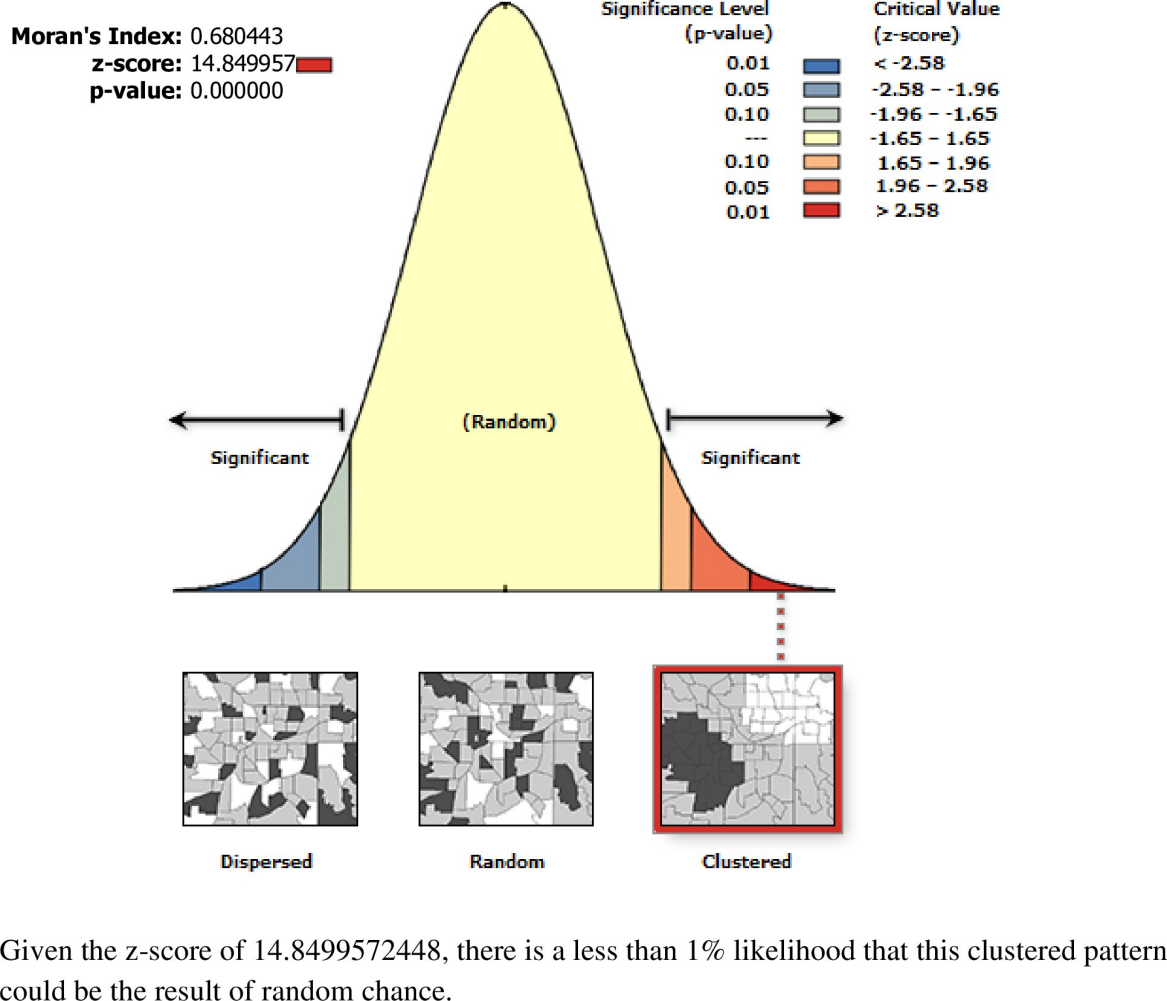
Spatial autocorrelation analysis of bottle feeding among children aged 0–23 months in Ethiopia.

**Fig 2:**
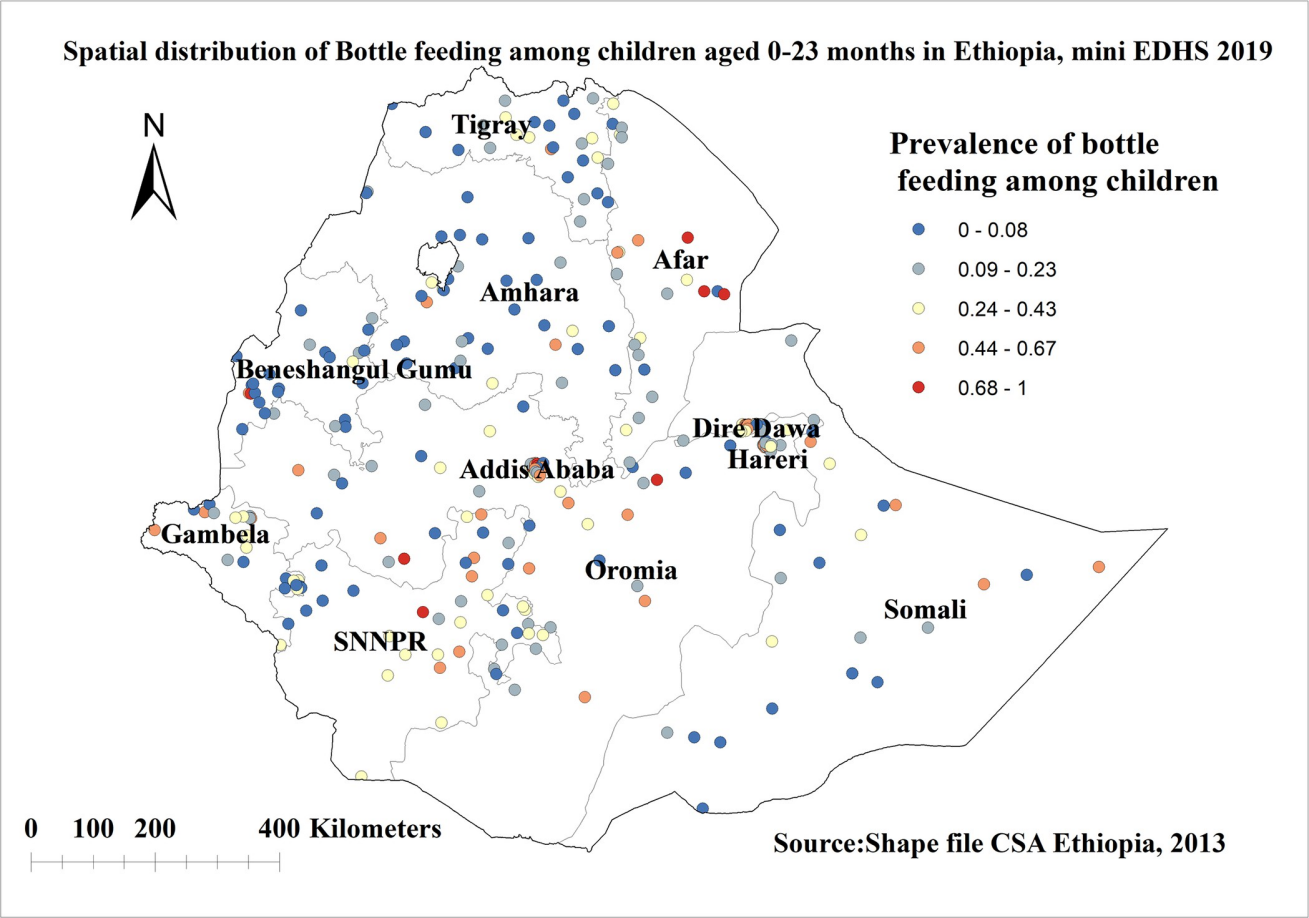
Spatial distribution of bottle feeding among children aged 0–23 months in Ethiopia, shape file source: Central Statistical Agency 2013, URL: https://africaopendata.org/dataset/ethiopia shape files. Map produced using ArcGIS version 10.7.

The spatial patterns of bottle feeding in Ethiopia revealed a significant spatial heterogeneity across the country over regions, which was found to be non-random with Global Moran’s I value of 0.68 with (p< 0.0000) (**Fig 1**). The incremental autocorrelation result revealed statistically significant z-scores at a peak distance of 203.187 km 10.53 (distances; Z-score) for bottle feeding. A total of 305 clusters were considered for the spatial analysis of bottle feeding. Each point on the map represents one enumeration area with a proportion of bottle feeding in each cluster. The red color indicates areas with a high proportion of bottle feeding observed in Addis Ababa, Dire Dawa, Harari, Afar, and northern SNNPR regions of Ethiopia whereas the blue color indicates EAs with a lower proportion of bottle feeding which was observed in Amhara, Benishangul Gumuz, Somalia, Tigray, western SNNPR, and Gambella regions of Ethiopia (**Fig 2**).

### Hotspot and cold spot analysis of bottle feeding in Ethiopia

**Fig 3:**
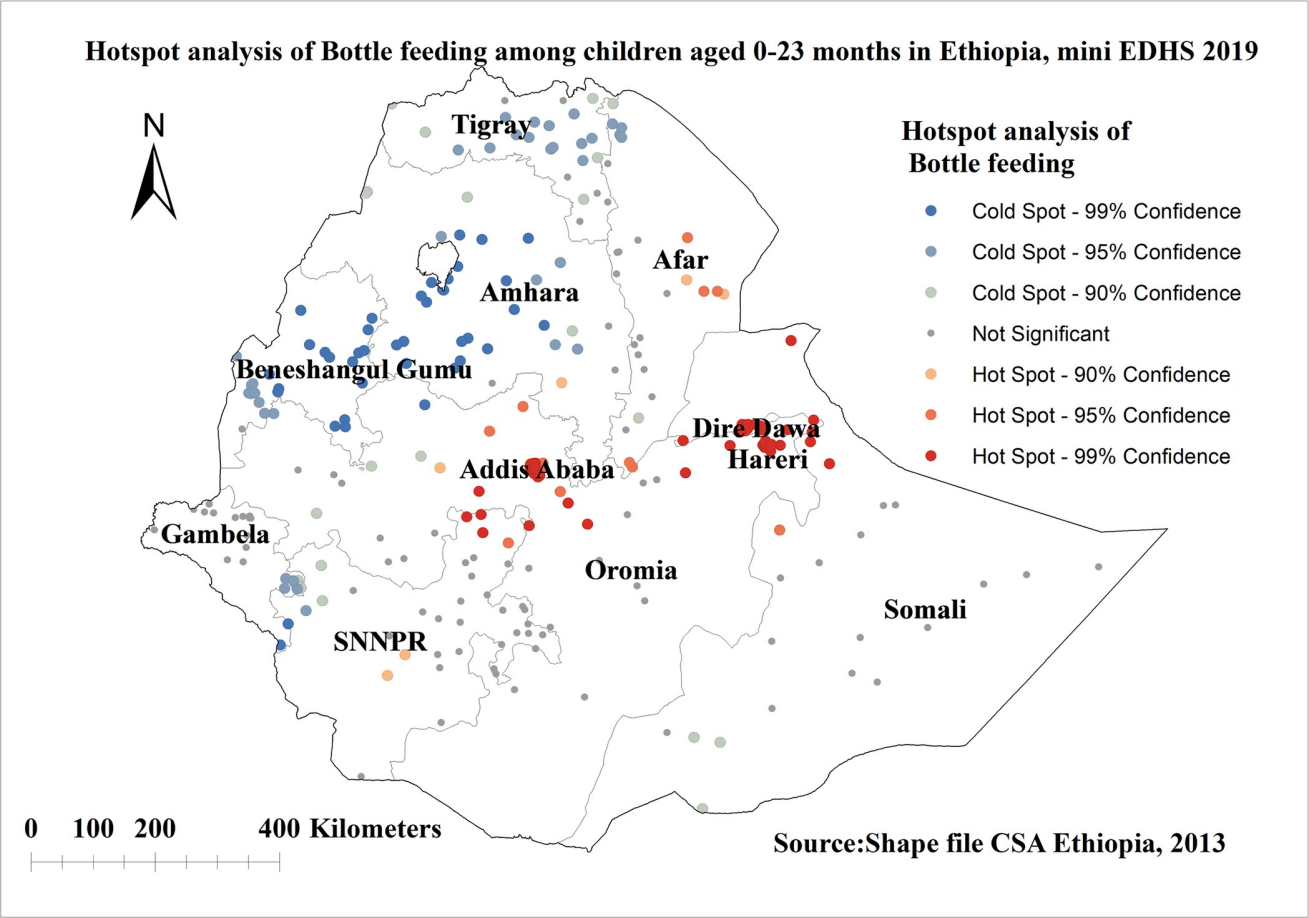
Hot spot analysis of bottle feeding among children aged 0–23 months in Ethiopia, shape file source: Central Statistical Agency 2013, URL: https://africaopendata.org/dataset/ethiopiashape files. Map produced using ArcGIS version 10.7.

The red color indicates regions with significant hotspot areas (areas with high rates of bottle feeding), which were found in the Addis Ababa, Dire Dawa, Harari, and Afar regions. The blue color indicates areas/regions with significantly lower rates of bottle feeding (cold spot areas), which were observed in Amhara, Tigray, Benishangul gumuz, and in small parts of SNNPR and Gambella regions (**Fig 3**).

### Kriging interpolation analysis of bottle feeding in Ethiopia

**Fig 4:**
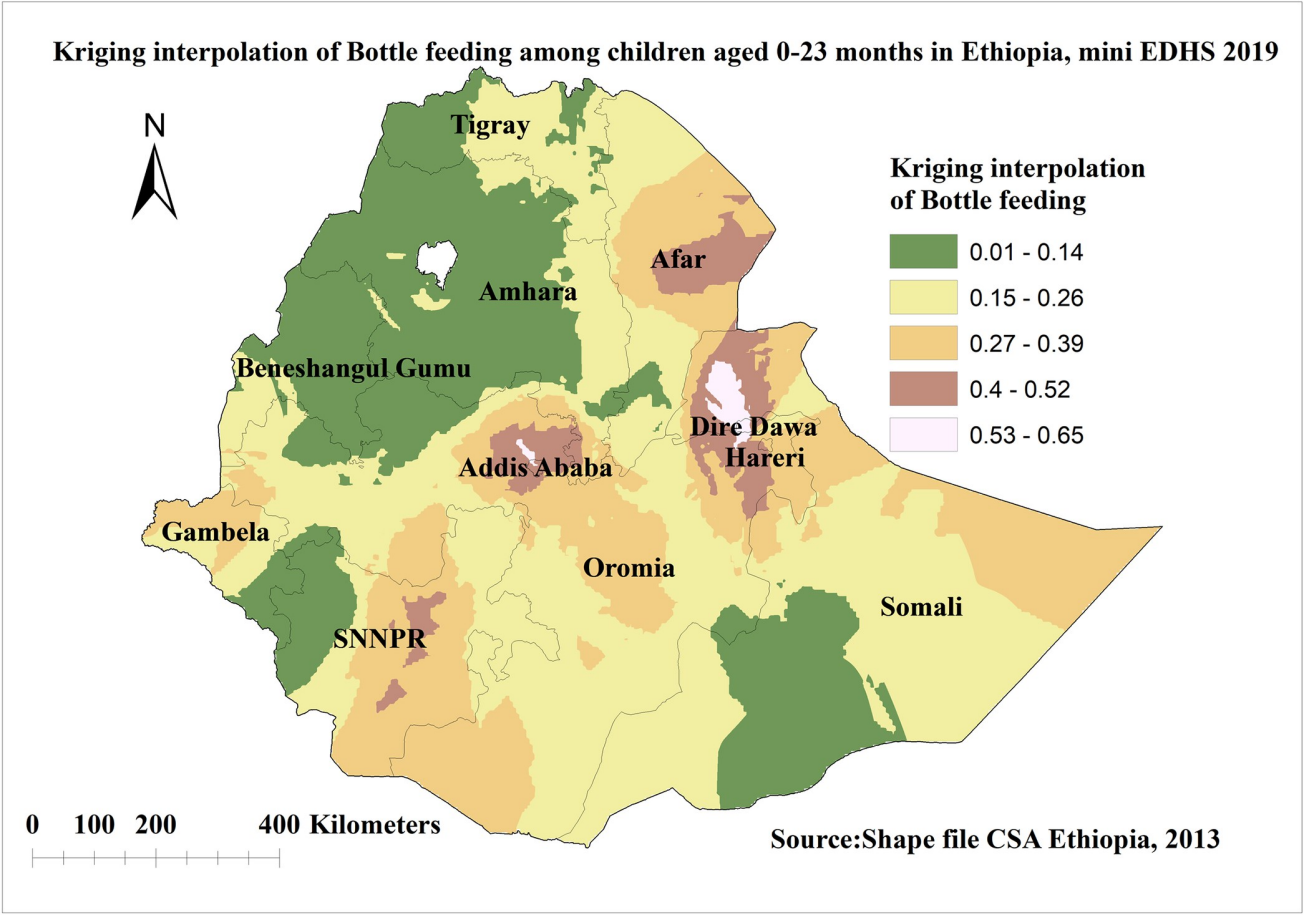
Kriging interpolation of bottle feeding among children aged 0–23 months in Ethiopia, shape file source: Central Statistical Agency 2013, URL: https://africaopendata.org/dataset/ethiopia shape files. Map produced using ArcGIS version 10.7.

The proportion of bottle feeding in the unsampled area was predicted using ordinary Kriging interpolation. The highest predicted areas of bottle feeding among children aged 0–23 months were moderate and ranging from 53% to 65% and located in Dire Dawa, Addis Ababa, and Harari regions. Whereas the lower predicted area was seen in, Amhara, Benishangul Gumuz, Somalia, Tigray, and some parts of SNNPR and Gambella region regions and ranges from 1% to 14% (**Fig 4**).

### Spatial scan statistical analysis of bottle feeding in Ethiopia

**Fig 5:**
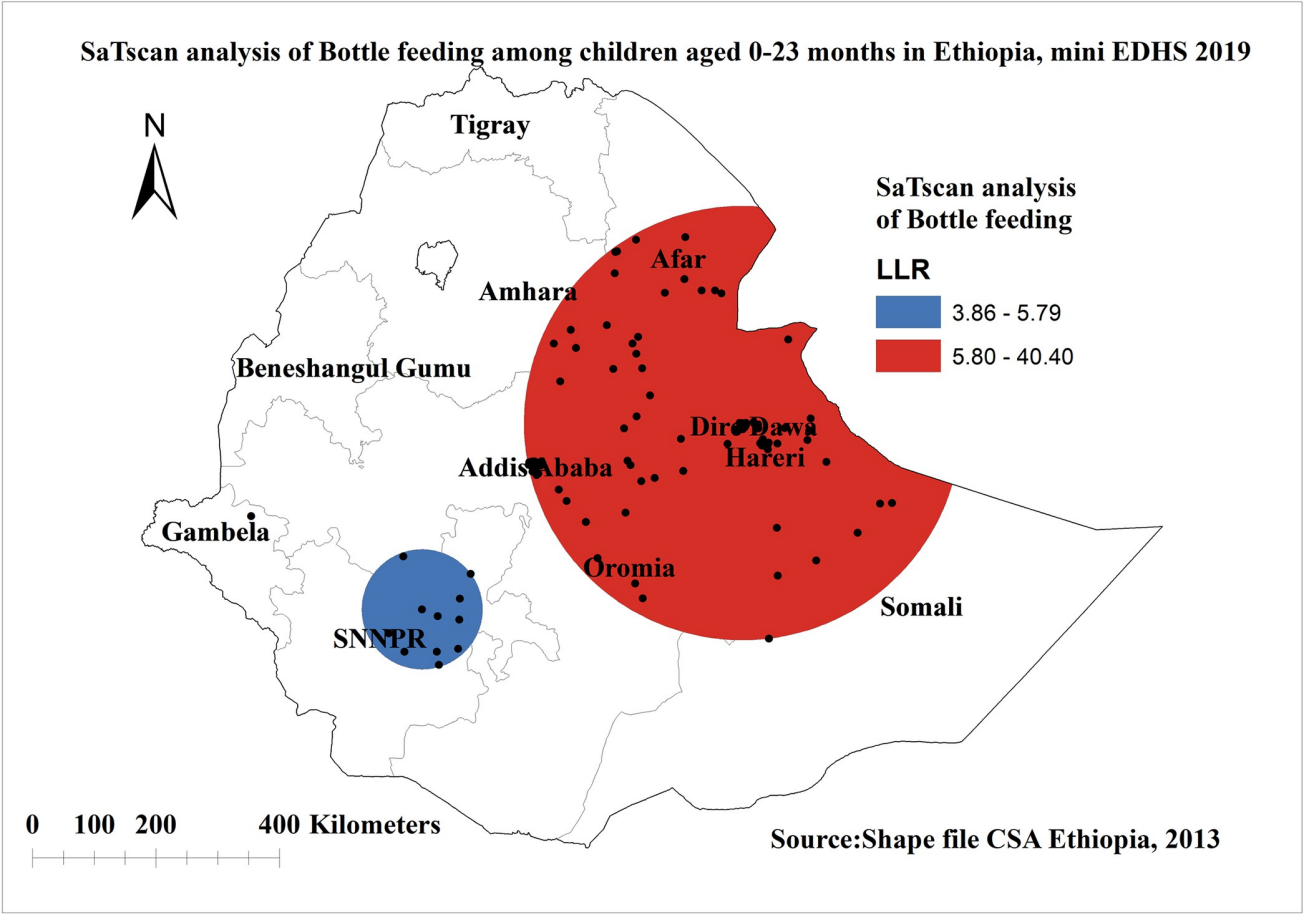
Spatial scan statistics analysis of bottle feeding among children aged 0–23 months in Ethiopia, shape file source: Central Statistical Agency 2013, URL: https://africaopendata.org/dataset/ethiopia shape files. Map produced using ArcGIS version 10.7.

The Bernoulli model was used to perform purely spatial analysis scanning for clusters with high rates. A total of 126 significant clusters of bottle feeding were specified. The primary cluster was found in Dire Dewa, Addis Ababa, Harari, and in some parts of Somalia, Oromia, Amhara, and Afar regions of Ethiopia at 9.614701 N, 41.829121 E with a 353.02 km radius, with a relative risk of 2.05 and log-likelihood ratio (LLR) of 40.39 at a p-value< 0.0000(**Fig 5**).

### The fixed effect analysis result

In the final selected model variables such as the educational status of women, the wealth index of the household, the age of the child, and the plurality of birth were significant predictors of bottle-feeding among children aged 0–23 months in Ethiopia.

Women with secondary or higher education were 2.09 [AOR = 2.09; 95%CI; 1.31, 3.32] times more likely to bottle feed their children than women with no formal education. The odds of having bottle feeding among children from middle and rich wealth status families were 2.14 and 2.30 times more likely to have bottle feeding as compared to a child from poor wealthy families [AOR = 2.14; 95%CI; 1.37, 3.34] and [AOR = 2.30; 95%CI; 1.46, 3.63] respectively. Children aged 6–11 months and 12–23 months were 5.69 and 3.87 times more likely to be bottle-fed than children aged 0–5 months, respectively [AOR = 5.69; 95%CI; 3.70, 8.74] and [AOR = 3.87; 95%CI; 2.64, 5.66] respectively. Multiple births were 8.06 times more likely to have bottle feeding than those who were single births [AOR = 8.06; 95%CI; 2.87, 22.58] (**Table 3**).

**Table 3:** Multilevel multivariable analysis of factors associated with bottle feeding among children aged 0–23 months in Ethiopia, 2019 mini EDHS.

Variables	categories	Null model	Model I	Model II	Model III
			AOR [95% CI]	AOR [95% CI]	AOR [95% CI]
Age of women	15–24		1.00		1.00
	25–34		1.13 [0.79, 1.61]		1.09 [0.76, 1.56]
	35–49		0.99 [0.58, 1.69]		0.95 [0.55, 1.63]
Marital status	Married		1.23 [0.67, 2.26]		1.17 [0.64, 2.16]
	Not married		1.00		1.00
Women education status	No education		1.00		1.00
	Primary		1.04 [0.75, 1.44]		0.98 [0.70,1.37]
	Secondary and higher		2.32 [1.47, 3.64]		2.09 [1.31, 3.32]***
Age of the child(in months)	0–5		1.00		1.00
	6–11		5.70 [3.70, 8.78]		5.69 [3.70, 8.74]***
	12–23		3.93 [2.69, 5.76]		3.87 [2.64, 5.66]***
Place of delivery	home		1.00		1.00
	Institution		0.97 [0.68, 1.37]		0.91 [0.64, 1.30]
Plurality of birth	Single		1.00		1.00
	Multiple		8.00 [2.87, 22.27]		8.06[2.87, 22.58]***
Sex of child	Male		1.00		1.00
	Female		0.83 [0.63, 1.08]		0.82[0.63, 1.08]
Parity	Primi		1.00		1.00
	Multiparous		1.14 [0.79, 165]		1.15 [0.79,1.67]
	Grand multiparous		0.77 [0.45, 1.31]		0.79 [0.46, 1.34]
Household head	Male		1.00		1.00
	Female		0.83 [0.63, 1.08]		1.04 [0.69, 1.55]
Wealth index	Poor		1.00		1.00
	Middle		2.25 [1.47, 3.43]		2.14 [1.37, 3.34]***
	Rich		2.79 [1.85, 4.20]		2.30 [1.46, 3.63]***
**Community level variables**					
Residence	Rural			0.61 [0.35, 1.07]	0.76 [0.40, 1.42]
	Urban			1.00	1.00
Community level poverty	Low			1.00	1.00
	High			0.45[0.27, 0.75]	0.74 [0.41, 1.33]
Community level of women’s education	Low			1.00	1.00
	High			1.56 [0.97, 2.52]	1.31 [0.75, 2.26]
Region	Large central			1.00	1.00
	Small peripheral			1.66 [0.91, 3.02]	1.79 [0.92, 3.47]
	Metropolitans			1.98 [0.97, 4.04]	1.77 [0.80, 3.91]

* = P-value < 0.05, ** = Pvalue < 0.01, *** = Pvalue < 0.001.

AOR = adjusted odds ratio; CI = confidence interval.

## Discussion

The study attempted to assess the spatial variation and determinants of bottle feeding among children aged 0–23 months using the recent demographic and health survey data of Ethiopia. The prevalence of bottle feeding among children aged 0–23 months was 21.52% which shows an 8% increment from 2016 EDHS indicating the need to reevaluate interventions to reduce bottle feeding and improve appropriate breastfeeding practices among mothers by the concerned bodies. This increasing rate of bottle-feeding practice may cause developmental problems, morbidity, and mortality in children [10,13]. This finding was consistent with studies conducted in Ethiopia [21,42]. However, it’s lower than studies conducted in Namibia [22], Indonesia [43], and Sudan [44]. Also, the finding was higher than previous studies done in Ethiopia [4], and India [45]. The possible explanation for this disparity could be attributed to differences in study area, socio-demographic status of participants, socio-economic status of participants, knowledge of mothers towards bottle feeding, and variation in cultural child feeding practices across different settings.

The spatial analysis revealed that the spatial pattern of bottle feeding among children was significantly varied across the country. The results of hot spot analysis revealed bottle-feeding hotspots were situated in Addis Ababa, Dire Dawa, Harari, and Afar regions, while the cold spot was observed in Amhara, Tigray, Benishangul gumuz, and in small parts of SNNPR and Gambella regions. Studies also indicated that metropolitan regions of Ethiopia may have a higher likelihood of bottle feeding than living in small peripheral and large central regions[4]. The possible reason might be that living in metropolis regions increases the chance of having access to breastfeeding alternatives which may in turn increase the chance of bottle-feeding practices. Besides the included community level factors (residence, community level poverty, community level of women’s education, region) were not significant predictor of bottle-feeding practice in the multilevel analysis.

As per the finding of this study, the likelihood of bottle-feeding increases with increasing child’s age. This is supported by a study conducted in Ethiopia [21,46], Nambia [22], Indonesia [43], Pakistan [23,24], and Uganda [47]. The fact is that bottle use is mostly associated with the start of complementary feeding, which is commonly initiated as the child’s age progresses.

The educational status of women was another significant variable for bottle feeding. Women with secondary and higher educational status have high likely to bottle feed their children as compared to women having no formal education. This finding was in line with studies done in Nigeria [25], Indonesia [43], India [26], Nambia [22], and Pakistan [23]. The possible reason might be that educated mothers are more likely to be employed and they may not have enough time to breastfeed their children which may force them to choose bottle feeding over breastfeeding [21].

Mothers from middle and rich wealth indices were more likely to practice bottle feeding than mothers from poor households. This finding was consistent with previous studies done in Nigeria [25], Nambia [22], India [26], Pakistan [23], and Indonesia [43]. The possible reason might be that women from the middle and rich wealth index may have the ability to purchase foods for their children and use bottle feeding as an alternative to breastfeeding [48]. Bottle feeding was more common in twin births than in single births. This is in line with a study conducted in Ethiopia [4], and Japan [49]. The fear of the mothers in producing enough milk for their twins, as well as competition for nutritional intake among the child may push mothers to breastfeed their children [20].

The study’s main strength was that it used nationally representative data with a large sample size and used an appropriate statistical approach to accommodate the data’s hierarchical nature. However, this study had limitations in that the cross-sectional nature of the data makes it impossible to infer causality between the independent and dependent variables. Furthermore, because it was a secondary data analysis, the data did not include information about some predictor variables of bottle feeding.

## Conclusion

Bottle feeding practice was found to be spatially clustered in Ethiopia. Education, wealth index, parity, and child’s age were significant predictors of bottle feeding. Hotspot areas of bottle feeding were observed in Addis Ababa, Dire Dawa, Harari, and Afar regions. Special attention should be directed towards mothers residing in hotspot areas, educated mothers, mothers of multiple births, and mothers from rich households through community education programs focused on child feeding practices to reduce the practice of bottle-feeding in Ethiopia.

## Abbreviations

AOR: Adjusted Odds Ratio
CI: Confidence Interval
COR: crude odds ratio
DHS: Demographic and Health Survey
EAs: Enumeration Areas
EMDHS: : Ethiopian mini Demographic and Health Survey
ICC: Intra Class Correlation
IYCF: infant and young child feeding
LLR: Log-likelihood ratio
MOR: Median Odd Ratio
PCV: Proportional Change in Variance
SNNP: Southern nation nationalities of people
VIF: Variance inflation factor
WHO: World Health Organization
